# Responses of Restored Vegetation Communities, Soil Properties, and Microbial Composition to Different Fertilization Treatments in an Alpine Mining Area

**DOI:** 10.3390/plants15040569

**Published:** 2026-02-11

**Authors:** Zhongyang Yu, Changhui Li, Mingchun Yang, Guoning Jing, Jianing Li, Jianli Wu

**Affiliations:** 1 College of Agriculture and Animal Husbandry, Qinghai University, Xining 810016, China; yb220909000055@qhu.edu.cn (Z.Y.);; 2 Veterinary Medicine and Academy of Animal Science, Qinghai University, Xining 810016, China

**Keywords:** alpine mining area, physicochemical properties, soil microbial biomass, soil microorganisms

## Abstract

As a typical ecologically degraded mining area, the Jiangcang Mine in Qinghai is characterized by severely depleted soil nutrients and reduced biodiversity, making scientifically grounded soil-amelioration measures urgently necessary to facilitate vegetation reconstruction and enhance soil ecological functions. To determine the optimal fertilization rate, we conducted a two-factor randomized block experiment over an approximately two-year period after plant sowing, using pelletized organic fertilizer and sheep manure as the primary amendments, with three replicates per treatment, and with application rates selected based on commonly used ranges in alpine grassland restoration and the availability of local organic resources in the mining area. Sheep-manure treatments were set at three levels at 0 (S0), 20 (S1), and 40 (S2) kg·m^−2^, while pelletized organic fertilizer was applied at three rates at 0.0 (F0), 1.5 (F1), and 3.0 (F2) kg·m^−2^. The combination of the two factors resulted in nine treatments: S0F0, S0F1, S0F2, S1F0, S1F1, S1F2, S2F0, S2F1, and S2F2. The results showed that fertilization significantly improved vegetation height, canopy cover, plant density, and aboveground biomass, with the strongest promotive effects observed under S2F2 and S2F1. Compared with other treatments, S2F2 markedly increased soil moisture content, pH, soil organic matter, available nitrogen, available phosphorus, as well as total nitrogen, 6.96-, 2.91-, 1.70-, 5.04-, 2.51-, and 3.91-fold relative to the control, respectively. The S0F2 treatment significantly enhanced bacterial Observed Richness, Shannon, and Chao1 indices, as well as simultaneously increasing fungal Observed Richness and Chao1 index. Vegetation height and density exhibited the strongest positive correlation under S2F1, whereas vegetation cover and aboveground biomass were most strongly correlated under S2F2. A gray relational analysis performed on 15 indicators ranked S2F0 as having the highest relational degree and comprehensive score, followed by S2F2 and S2F1. In summary, the combined application of approximately 40 kg·m^−2^ of sheep manure without pelletized organic fertilizer showed the highest comprehensive restoration performance under the experimental conditions of this alpine mining area.

## 1. Introduction

The Jiangcang Coal Mine is the largest mining area in Qinghai Province, with an average elevation of approximately 3800 m. The region is characterized by long cold seasons, low oxygen availability, and short plant growing periods [1]. Prolonged coal mining activities have caused severe damage to the original alpine grassland ecosystem, giving rise to a series of environmental problems, including extensive areas requiring rehabilitation, extremely poor soil conditions, elevated soil pH, slow natural recovery rates, nutrient deficiency for plant growth, declines in biodiversity, and increasingly pronounced ecosystem degradation [2]. In addition, the highly variable climate, extreme ecological fragility, and continuous mining disturbances have further exacerbated soil degradation. Consequently, ecological restoration of the Jiangcang mining area has become an urgent priority. Given the difficulty of natural recovery, artificial intervention has emerged as the most effective approach. Establishing artificial grasslands can initiate vegetation recovery and subsequently facilitate ecological restoration through community succession [3]. However, due to the high elevation, low mean annual temperature, and harsh environmental conditions, newly established vegetation systems often lack stability. Although previous studies have shown that topsoil covering can effectively promote vegetation restoration in mining areas, this approach is limited by high cost and logistical difficulties associated with transporting soil from other locations, making it unsuitable for large-scale application [4].

To address these limitations, this study applies organic fertilizers to ameliorate mine spoil and establish artificial vegetation. Fertilization can enhance soil fertility and soil enzyme activities in mining areas [5], as well as increase the supply of essential nutrients. As one of the most widely used soil amendments, organic fertilizer provides a comprehensive nutrient profile and releases nutrients slowly, making it well suited for the slow recovery process typical of high-altitude mining environments. Moreover, because organic fertilizers generate minimal environmental pollution, they are appropriate for ecological restoration in mine sites [6,7]. Considering the remoteness of the mining area and the high transport cost of commercial organic fertilizers, local resources offer a practical alternative. Local herders primarily raise sheep and cattle, and sheep trampling results in compressed manure mixed with surface soil, forming a type of naturally processed fertilizer known as “sheep-panel manure.” Although this material is abundant, its utilization rate remains low. Due to its readily available raw materials, ease of transport, and strong fertilizing effect, sheep-panel manure could serve as a priority amendment in high-altitude mining areas [8]. However, because it is not fully decomposed, its initial nutrient release rate is relatively slow. The low annual temperatures of the mining area further impede decomposition, as sheep-panel manure typically requires temperatures around 50 °C to fully mature. As a result, complete decomposition takes 2–3 years longer than conventional composting, but this slow-release pattern matches the long-term recovery needs of mining ecosystems and facilitates the formation of fertile topsoil required for sustained vegetation growth [9]. In contrast, pelletized organic fertilizers are fully decomposed during industrial processing and are typically enriched with readily available nutrients and beneficial microbial inoculants. Owing to their rapid nutrient release, standardized quality, and ease of application, pelletized organic fertilizers are widely used in ecological restoration practices, particularly to support early-stage vegetation establishment under stressful environmental conditions. In high-altitude mining areas, where low temperatures constrain organic matter decomposition, pelletized organic fertilizers can rapidly alleviate nutrient limitation and promote initial plant growth. Therefore, pelletized organic fertilizer was included in this study both as a commonly used restoration amendment and as a complementary fertilizer to sheep-panel manure, enabling an evaluation of their individual and combined effects on vegetation recovery and soil–microbial processes [10].

Soil physicochemical properties are key indicators for evaluating soil condition and the effectiveness of soil amendments [11]. The addition of livestock manure significantly increases soil organic matter, nitrogen, and phosphorus inputs. Soil organic matter plays a critical role in improving soil physical stability, nutrient storage, and carbon sequestration. Increased organic matter also enhances microbial diversity and relative abundance, stimulates microbial activity, and accelerates nutrient cycling, thereby improving soil physicochemical properties and promoting vegetation growth [12]. Soil pH, alkali-hydrolyzable nitrogen, and available phosphorus also influence soil conditions and plant development [13]. Therefore, this study uses soil physicochemical parameters as the main indicators to evaluate the amelioration of mine spoil. Soil microbial biomass (SMB) is another important indicator of natural soil fertility. It is closely linked to the synthesis and decomposition of soil organic matter and responds sensitively to environmental changes [14,15]. Microorganisms are essential to soil health, as they decompose organic substrates, release nutrients, and supply plants with essential elements. Although microbial biomass carbon (MBC) accounts for only 1–4% of soil organic matter [16], this small fraction plays a central role in nutrient uptake, transformation processes, quality regulation, and energy storage. Microbial biomass forms complex networks of interactions with nitrogen, carbon, sulfur, and phosphorus, maintaining soil chemical balance and biodiversity. Microbial biomass nitrogen serves as a key source of available N, and its concentration directly reflects the soil’s nitrogen supply capacity [17]. Fertilization can stimulate microbial growth and reproduction, increase microbial abundance and diversity, and modify community structure [18], thereby regulating soil physiological and biochemical processes, improving soil nutrient availability, and enhancing vegetation restoration. Soil bacteria, which dominate soil microbial communities, participate in most nutrient transformation processes [19,20]. Soil total phosphorus and available phosphorus strongly affect bacterial communities, whereas available nitrogen and pH are considered major environmental drivers of fungal community composition [21], as well as important indicators of soil ecosystem status.

Therefore, this study investigates the responses of soil fungal and bacterial communities to the application of sheep-panel manure and pelletized organic fertilizer in a high-altitude mining area using high-throughput sequencing. Vegetation characteristics, soil physicochemical properties, soil microbial biomass, and microbial community diversity were measured under sheep manure application, pelletized organic fertilizer addition, and their combined treatments. By examining the interactions among vegetation traits, soil properties, microbial biomass, and microbial community structure under different treatments, this study provides theoretical support for ecological restoration in high-altitude mining regions.

## 2. Results

### 2.1. The Influence of Different Fertilization Patterns on Plant Characteristics

**Figure 1:**
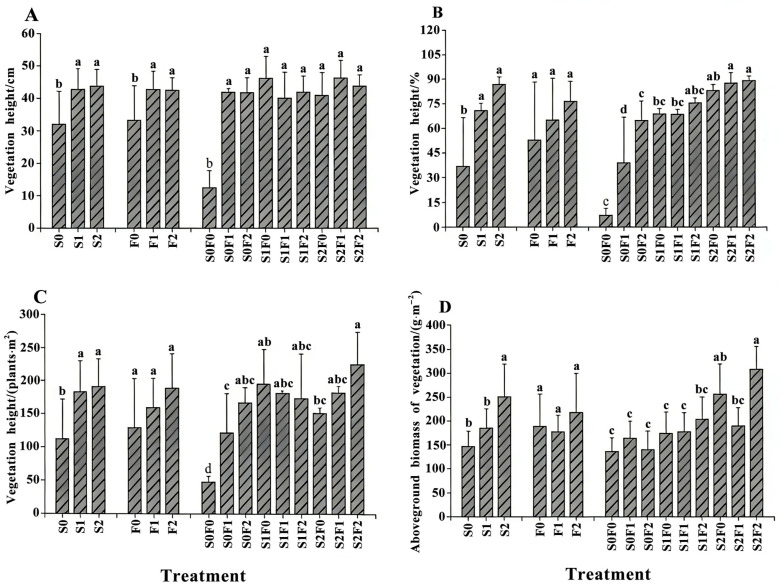
Changes in vegetation community characteristics under different fertilization treatments. Note: (**A**) Vegetation height; (**B**) Vegetation coverage; (**C**) Vegetation density; (**D**) Aboveground biomass of vegetation. S0, S1, and S2 represent the main effects of sheep-panel manure application rates (0, 20, and 40 kg·m^−2^, respectively), averaged across all levels of pelletized organic fertilizer. F0, F1, and F2 represent the main effects of pelletized organic fertilizer application rates (0, 1.5, and 3.0 kg·m^−2^, respectively), averaged across all levels of sheep-panel manure. S0F0 denotes the unfertilized control treatment without either amendment. Different lowercase letters indicate significant differences among treatments at *p* < 0.05, the same as below.

Overall, the different fertilization treatments significantly improved the community structure of the artificial grassland in the mining area (*p* < 0.05) (Table S1, Figure 1). Fertilization markedly increased vegetation height, canopy cover, plant density, and aboveground biomass (expressed as dry weight, g·m^−2^), with the combined application of sheep-panel manure and pelletized organic fertilizer showing the most pronounced effects. Compared with S0, vegetation height, canopy cover, and plant density all increased significantly under the S1 and S2 treatments (*p* < 0.05), while aboveground biomass was significantly higher under S2 than under all other treatments (*p* < 0.05). Relative to F0, vegetation height increased significantly under F1 (*p* < 0.05), whereas plant density and canopy cover reached their highest values under F2, both significantly exceeding those of F0 (*p* < 0.05). Compared with the unfertilized control (S0F0), vegetation height was significantly enhanced under the combined S2F1 treatment, while canopy cover, plant density, and aboveground biomass were significantly higher under S2F2 (*p* < 0.05).

### 2.2. The Influence of Different Fertilization Patterns on Soil Physicochemical Properties

**Figure 2:**
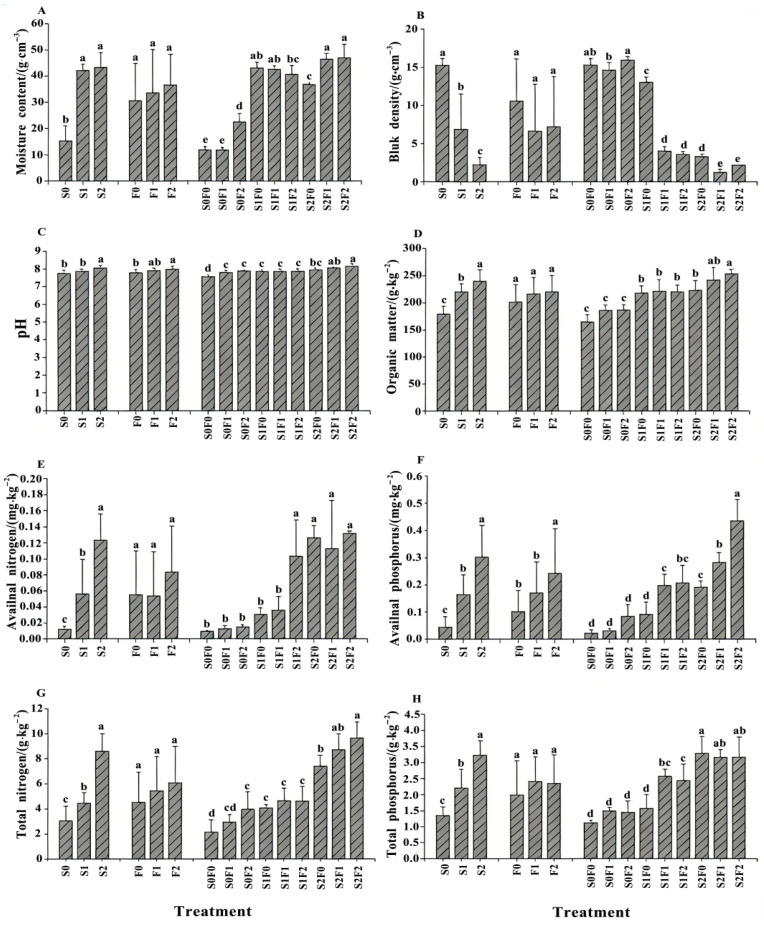
Changes in soil physicochemical properties under different fertilization treatments. Note: (**A**) Moisture content; (**B**) Bluk density; (**C**) Soil pH; (**D**) Organic matter; (**E**) Availnal nitrogen; (**F**) Availnal phosphorus; (**G**) Total nitrogen; (**H**) Total phosphorus. Different lowercase letters indicate significant differences among treatments at *p* < 0.05.

Significant differences in soil physicochemical properties were observed among the fertilization treatments (Table S2, Figure 2). Fertilization notably increased soil moisture content, total and available nutrient concentrations, and soil organic matter, while reducing bulk density. Soil total nitrogen was significantly higher under the S2 and F2 treatments than under all other levels (*p* < 0.05). Total phosphorus was significantly higher under S2 and F1 (*p* < 0.05). For available nitrogen, available phosphorus, soil moisture content, pH, and organic matter, the patterns under sheep-panel manure and pelletized organic fertilizer followed S2 > S1 > S0 and F2 > F1 > F0, respectively. Within the organic fertilizer treatments, available phosphorus and pH were significantly higher under F2 than under F0 (*p* < 0.05), while differences among other treatments were not significant. In the combined treatments, soil total nitrogen, total phosphorus, available nitrogen, available phosphorus, soil moisture content, pH, and organic matter all exhibited increasing trends with higher application rates. Total nitrogen and total phosphorus reached their maximum values under S2F2 and S2F0, respectively, at 9.86 g·kg^−1^ and 3.21 g·kg^−1^. Available nitrogen, available phosphorus, moisture content, pH, and organic matter all reached their highest levels under S2F2. These results indicate that increasing the application rates of sheep-panel manure, pelletized organic fertilizer, and their combined use can effectively improve the physicochemical properties of spoil soils in mining areas.

### 2.3. The Influence of Different Fertilization Patterns on Soil Microbial Biomass

**Figure 3:**
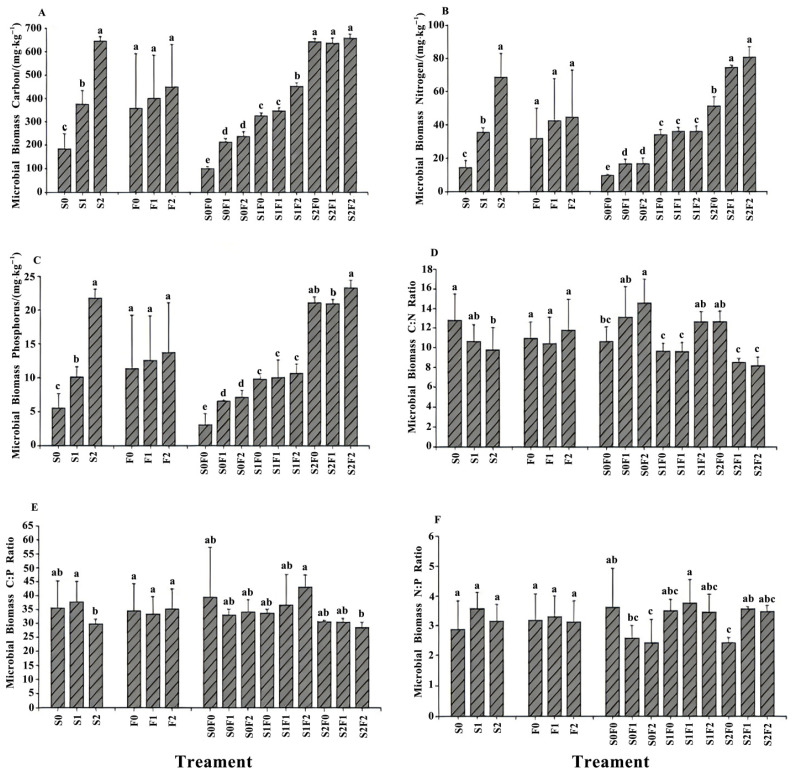
Changes in soil microbial biomass under different fertilization treatments. Note: (**A**) Microbial biomass carbon (MBC); (**B**) Microbial biomass nitrogen (MBN); (**C**) Microbial biomass phosphorus (MBP); (**D**) Microbial biomass C:N ratio; (**E**) Microbial biomass C:P ratio; (**F**) Microbial biomass N:P ratio. Different lowercase letters indicate significant differences among treatments at *p* < 0.05.

Soil microbial biomass C:N ratios can reflect the long-term nitrogen storage capacity of the soil nutrient pool (Table S3, Figure 3). Under the sheep-panel manure treatments, the soil MBC/MBN ratio ranged from 9.77 to 12.77. Both MBC/MBN and MBC/MBP were significantly lower under the S2 treatment compared with S0 (*p* < 0.05), whereas MBN/MBP showed no significant differences among treatments. Under the pelletized organic fertilizer treatments, none of the microbial biomass ratios differed significantly. In the combined treatments, soil MBC/MBN was significantly higher under S0F2 than under all other treatments (*p* < 0.05), while no significant differences were observed among the remaining treatments. Soil MBC/MBP was significantly lower under S2F2 than under all other treatments (*p* < 0.05). Except for S1F2, all other treatments showed higher MBC/MBP ratios than the untreated control (S0F0). Soil MBN/MBP was significantly lower under S0F2 and S2F0 compared with the other treatments, while no significant differences were observed among the remaining treatment combinations.

### 2.4. Effects of Nutrient Addition on Soil Microbial Communities

#### 2.4.1. The Influence of Different Fertilization Patterns on Soil Microbial Diversity

**Figure 4:**
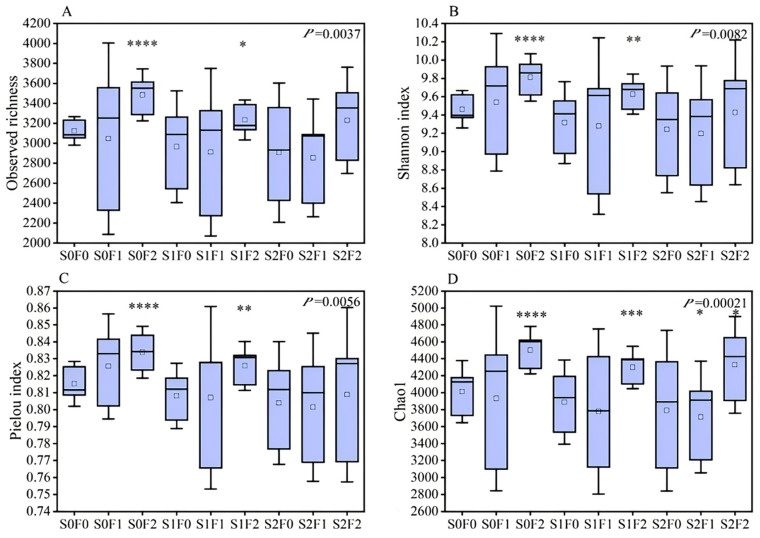
Changes in soil bacterial α-diversity under different fertilization treatments. Note: (**A**) Observed richness; (**B**) Shannon index; (**C**) Pielou index; (**D**) Chao1 index. Asterisks indicate significant differences among treatments (* *p* < 0.05; ** *p* < 0.01; *** *p* < 0.001; **** *p* < 0.0001).

Analysis of soil microbial α-diversity revealed clear differences among the fertilization treatments. For the bacterial community, Observed Richness showed extremely significant differences under the S0F2 treatment (*p* < 0.01) and significant differences under S1F2 (*p* < 0.05). Bacterial richness followed the order: S0F2 > S0F1 > S2F2 > S1F2 > S2F0 > S1F1 > S1F0 > S0F0 > S2F1. Species diversity, represented by the Shannon index, was also extremely significantly higher under S0F2 and S1F2 (*p* < 0.01). The S1F0 and S2F1 treatments showed lower Shannon values than the control (S0F0), whereas all other treatments exceeded the control. Species evenness, described by the Pielou index, differed extremely significantly under S0F2 and S1F2 (*p* < 0.01); S1F0 and S2F0 exhibited lower evenness than the control, while all other treatments were higher. The Chao1 index showed extremely significant increases under S0F2 and S1F2 (*p* < 0.01); only S2F1 was lower than the control, while all other treatments exceeded S0F0. Overall, these results demonstrate that S0F2 and S1F2 markedly enhanced bacterial α-diversity (Figure 4).

**Figure 5:**
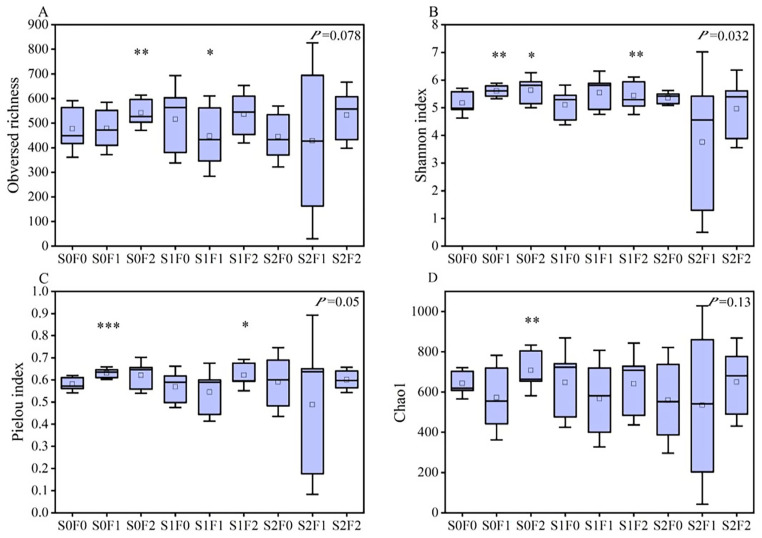
Changes in soil fungal α-diversity under different fertilization treatments. Note: (**A**) Observed richness; (**B**) Shannon index; (**C**) Pielou index; (**D**) Chao1 index. Asterisks indicate significant differences among treatments (* *p* < 0.05, ** *p* < 0.01, *** *p* < 0.001).

Observed Richness was extremely significantly higher under S0F2 (*p* < 0.01) and significantly higher under S1F1 (*p* < 0.05), with richness following the order: S2F1 > S1F2 > S2F2 > S1F0 > S0F2 > S0F0 > S1F1 > S0F1 > S2F0. For species diversity, represented by the Shannon index, S0F1 and S1F2 exhibited extremely significant increases (*p* < 0.01), while S0F2 showed a significant increase (*p* < 0.05). Species evenness, indicated by the Pielou index, was extremely significantly higher under S0F1 (*p* < 0.01) and significantly higher under S1F2 (*p* < 0.05), with S2F0 showing the highest evenness overall. The Chao1 index was extremely significantly elevated under S0F2 (*p* < 0.01), with S2F1 reaching the highest richness. Collectively, these findings indicate that S0F2 had the strongest positive effect on fungal α-diversity, followed by S1F2 and S0F1 (Figure 5).

#### 2.4.2. The Influence of Different Fertilization Patterns on Soil Microbial Community Structure

**Figure 6:**
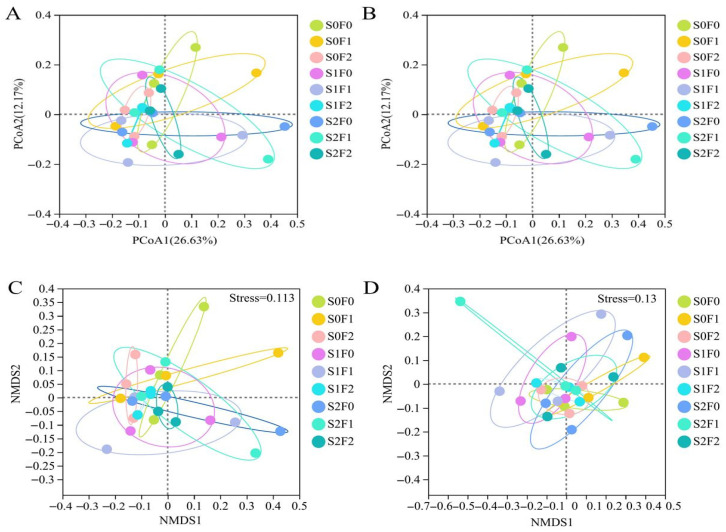
NMDS analysis and PCoA analysis based on Bray–Curtis distance matrix (**A**,**C**): Bacteria; (**B**,**D**): Fungi.

To examine differences in soil microbial β-diversity among the fertilization treatments, principal coordinate analysis (PCoA) based on Bray–Curtis distances was conducted. For the bacterial community (Figure 6A), PCoA1 and PCoA2 explained 26.63% and 12.17% of the total variation, respectively. The bacterial communities did not show clear separation among treatments, indicating relatively small evolutionary distances and high similarity in community composition. This pattern was further confirmed by non-metric multidimensional scaling (NMDS) based on Bray–Curtis distances (Figure 6C), which produced consistent clustering results.

For the fungal community (Figure 6B), the PCoA ordination was presented using the same coordinate axes (PCoA1 = 26.63% and PCoA2 = 12.17%) to facilitate direct visual comparison with bacterial communities. Similarly to bacteria, fungal communities did not exhibit distinct separation among fertilization treatments, suggesting limited differentiation in community composition. NMDS analysis (Figure 6D) confirmed the PCoA results and revealed comparable clustering patterns. Overall, these results indicate that fertilization did not fundamentally alter microbial community structure, and both bacterial and fungal assemblages remained tightly clustered across treatments.

#### 2.4.3. The Influence of Different Fertilization Patterns on the Composition of Soil Microbial Communities

**Figure 7:**
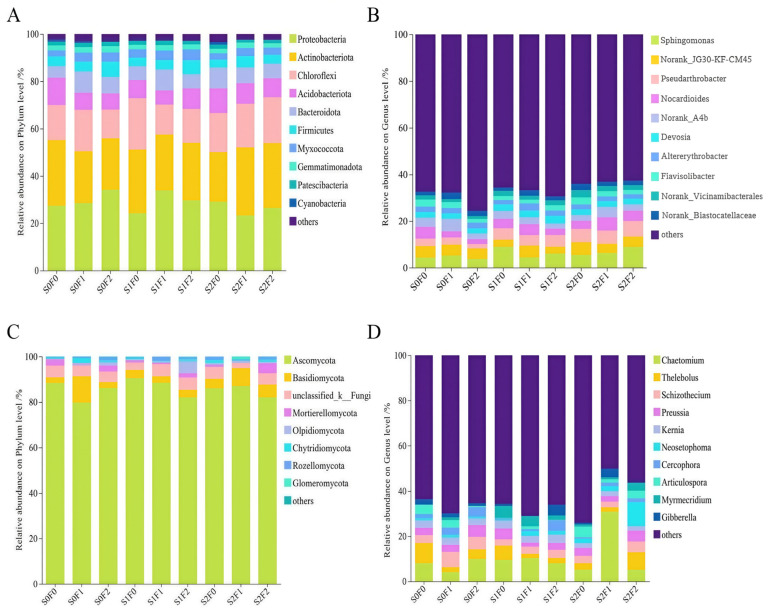
Composition of bacterial and fungal communities under different fertilization treatments. Note: (**A**): Phylum-level analysis of Bacteria; (**B**): Genus-level analysis of Bacteria; (**C**): Phylum-level analysis of Fungi; (**D**): Genus-level analysis of Fungi.

Based on the top ten most abundant taxa under each fertilization treatment, bar plots of bacterial and fungal community composition were generated according to relative abundance. At the bacterial phylum level (Figure 7A), the dominant phyla were Proteobacteria, Actinobacteriota, and Chloroflexi. Proteobacteria showed the highest relative abundances under the S0F2 and S1F1 treatments, reaching 34.16% and 33.88%, respectively. Actinobacteriota were most abundant under S2F1 (28.82%) and S0F0 (27.88%). At the genus level (Figure 7B), excluding unclassified taxa, Norank_ JG30-KF-CM45 exhibited relative abundances ranging from 3.06% to 5.49%, with the lowest value under S1F0 and the highest under S2F0. Pseudarthrobacter showed significantly higher relative abundance under S2F2 than under all other treatments, whereas S0F2 had the lowest proportion (1.85%).

At the fungal phylum level (Figure 7C), Ascomycota dominated the community, with relative abundances ranging from 79.75% to 90.58%, reaching the highest value under S1F0. The second most abundant phylum was Basidiomycota, which peaked under the S0F1 treatment (11.68%). At the genus level (Figure 7D), excluding unclassified taxa, the dominant genus was Chaetomium, which was significantly more abundant under S2F1 than under all other treatments, accounting for 33.77% of the fungal community.

### 2.5. Correlation Analysis of Vegetation, Soil, and Microbial Diversity

#### 2.5.1. Effects of Soil Physical and Chemical Properties and Microbial Biomass on Vegetation Characteristics

**Figure 8:**
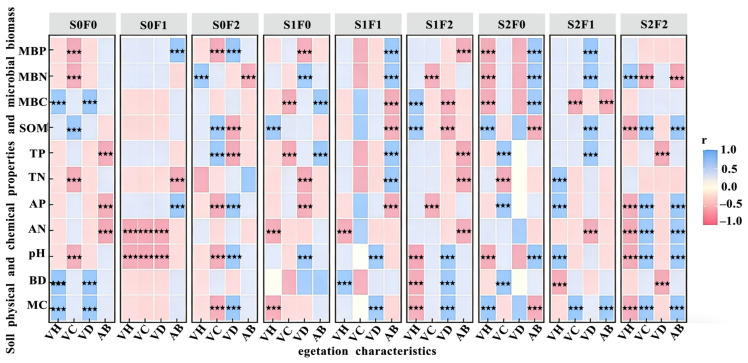
Effects of soil physical and chemical properties and microbial biomass on vegetation characteristics under different treatments. Note: VH: Vegetation height; VC: Vegetation cover; VD: Vegetation density; AB: Aboveground biomass; MC: Moisture content; BD: Bulk density; pH: Soil pH value; AN: Ammonium nitrogen; AP: Available phosphorus; TN: Total nitrogen; TP: Total phosphorus; SOM: Soil organic matter; MBC: Microbial biomass carbon; MBN: Microbial biomass nitrogen; MBP: Microbial biomass phosphorus; Blue represents positive correlation, red represents negative correlation; *** means *p* < 0.01.

The effects of soil physicochemical properties and microbial biomass on vegetation characteristics under different fertilization treatments in the alpine mining area are shown in Figure 8. Vegetation height (VH) exhibited the strongest positive correlation under the S2F1 treatment, whereas vegetation cover (VC) showed the strongest positive correlation under S2F2. Vegetation density (VD) was most strongly and positively correlated under S2F1, while aboveground biomass (AB) displayed the highest positive correlation under S2F2.

#### 2.5.2. Correlation Analysis Between Vegetation Community Characteristics, Soil Physicochemical Factors, Microbial Biomass and Its Stoichiometric Ratio, and Microbial Diversity

**Figure 9:**
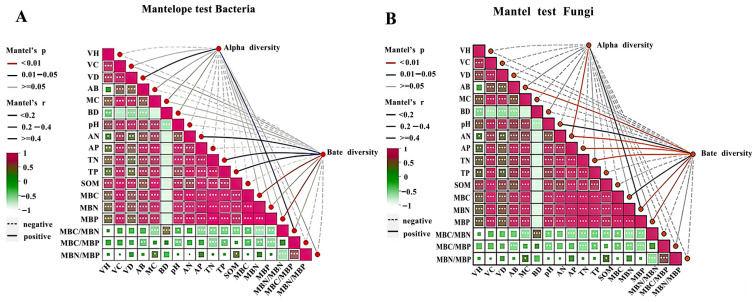
Mantel test of soil microbial α-diversity and β-diversity with environmental driving factors. Note: (**A**) is bacterial Mantel test, (**B**) is fungal Mantel test; VH represents plant height; VC represents vegetation coverage; VD represents vegetation density; AB represents aboveground biomass; pH represents pH; AN represents ammonium nitrogen; AP represents available phosphorus; TN represents total nitrogen; TP represents total phosphorus; SOM represents organic matter; lines of different colors represent significance; lines of different thickness indicate correlation; matrixes of different colors represent Pearson correlation. Asterisks indicate the significance level of Mantel tests: * *p* < 0.05, ** *p* < 0.01, and *** *p* < 0.001.

To identify the soil physicochemical factors that drive microbial community variation, Mantel tests were conducted to analyze the relationships between soil microbial α-diversity, β-diversity and vegetation traits, soil properties, microbial biomass, and microbial stoichiometric ratios. For the bacterial community (Figure 9A), vegetation density and the MBC/MBN ratio significantly influenced bacterial α-diversity (*p* < 0.05). Soil-available nitrogen, total nitrogen, and total phosphorus showed significant correlations with bacterial β-diversity (*p* < 0.05). Microbial biomass carbon (MBC) and microbial biomass phosphorus (MBP) were highly significantly correlated with bacterial β-diversity (*p* < 0.01).

For the fungal community (Figure 9B), microbial biomass nitrogen (MBN) significantly affected fungal α-diversity (*p* < 0.05), and soil available nitrogen exhibited a highly significant correlation with fungal α-diversity (*p* < 0.01). Microbial biomass carbon (MBC) and soil pH were significantly correlated with fungal β-diversity (*p* < 0.05). Aboveground biomass, available nitrogen, available phosphorus, total nitrogen, microbial biomass nitrogen (MBN), and microbial biomass phosphorus (MBP) all showed highly significant correlations with fungal β-diversity (*p* < 0.01), while microbial biomass carbon (MBC) was significantly correlated with fungal β-diversity (*p* < 0.05).

### 2.6. Evaluation of the Optimal Dosage for Application

First, the gray relational coefficients were weighted and calculated to obtain the final relational degree values, which were then used to rank the 15 evaluation indicators. The relational degree ranges from 0 to 1, with higher values indicating a stronger correlation with the reference sequence (mother sequence) and thus a higher evaluation level (Table 1). Comparative analysis of the nine treatment combinations revealed that the S2F0 treatment achieved the highest comprehensive score (relational degree: 0.936), followed by S2F2 and S2F1, both with relational degree values of 0.930 (Table 2).

## 3. Discussion

### 3.1. Effects of Different Fertilization Treatments on Plant Community Characteristics

Different restoration approaches can exert significant effects on plant growth, canopy cover, and biomass production [22]. Plant growth is a complex process easily influenced by external environmental conditions; factors such as soil moisture, temperature, light availability, and geographical location can all regulate plant development. Any alteration in these factors may lead to abnormal growth or even plant mortality [23]. In the Jiangcang mining area, the absence of a natural soil matrix means that direct seeding provides insufficient nutrients for plant growth, resulting in poor development and failure to achieve restoration goals. Therefore, this study adopted a restoration strategy that integrates fertilization with artificial vegetation establishment. Zhang Min et al. [24] reported that the application of organic fertilizer during artificial vegetation establishment in alpine mining areas can significantly increase vegetation height, canopy cover, and aboveground biomass, thereby improving plant growth and accelerating ecological restoration. Similarly, Cheng Huangxin et al. [25] found that sheep-panel manure effectively promotes vegetation growth during spoil–soil amelioration. Consistent with these findings, the results of this study show that increasing the application rate of sheep-panel manure led to significant improvements in vegetation height, canopy cover, plant density, and aboveground biomass, with the strongest responses observed under the S2 treatment (40 kg·m^−2^). In contrast, when pelletized organic fertilizer was applied alone, plant responses were irregular and exhibited no clear trend. Combined application treatments were the most effective overall. The S2F2 treatment (40 kg·m^−2^ sheep-panel manure + 3 kg·m^−2^ pelletized organic fertilizer) significantly enhanced vegetation cover, density, and aboveground biomass, whereas S2F1 (40 kg·m^−2^ sheep-panel manure + 1.5 kg·m^−2^ organic fertilizer) had the strongest effect on plant height. Overall, the mixed fertilization treatments produced the most pronounced benefits for vegetation recovery, followed by sheep-panel manure alone, while pelletized organic fertilizer alone showed relatively weak effects. These results align with the findings of Neldner Victor J et al. [26], who reported that sheep-panel manure outperformed commercial organic fertilizers in promoting vegetation recovery, and that combined application of the two produced greater improvements in canopy cover and aboveground biomass. This enhanced performance may be attributed to the broader nutrient profile and soil-ameliorating capacity of sheep-panel manure compared with pelletized organic fertilizer. Sheep-panel manure can supply a wider range of nutrients, improve soil aggregate structure, reduce soil compaction, and enhance aeration, thereby facilitating nutrient retention [27] and creating more favorable conditions for plant growth. Taken together, compared with the use of individual fertilizers, the combined application of sheep-panel manure and pelletized organic fertilizer provides a more complete nutrient supply and creates more suitable soil conditions for plant development, ultimately yielding the best restoration outcomes. However, it should be noted that vegetation responses in this study were evaluated primarily at the community structural level, and species-level composition was not explicitly quantified. At this early restoration stage, vegetation communities were largely dominated by sown species, and fertilization-induced increases in vegetation cover and biomass may enhance their competitive advantage, potentially constraining the establishment of colonizing species from surrounding alpine meadows.

### 3.2. Effects of Different Fertilization Treatments on Soil Physicochemical Properties

In the ecological restoration of mining areas, improving soil physicochemical properties and nutrient status plays a pivotal role in facilitating ecosystem recovery. Due to the inherently high bulk density of mine spoil, materials with abundant pore structures—such as humic acid [28] and straw-derived biochar [29]—are often required to enhance soil aeration, reduce compaction, and fill coarse pores, thereby improving water-holding capacity. Manure amendments are also widely used because they are rich in nutrients and natural binding agents that enhance soil structure and function [30,31]. According to Liu YaNan et al. [32], pelletized organic fertilizer can significantly influence soil total nitrogen and organic matter content. In the present study, pelletized organic fertilizer at the F2 level (3 kg·m^−2^) significantly affected soil pH and available phosphorus, but had no significant effect on other physicochemical properties. This may be attributed to the slow decomposition rate of pelletized organic fertilizer due to its granulation process, which may delay its impact on soil physical structure, especially in the short term. It may also reflect the inherently slow soil-forming processes in high-elevation, cold, and precipitation-rich mining environments [33]. Previous studies [34] have shown that applying an appropriate amount of well-decomposed sheep manure can increase soil nitrogen, phosphorus, and organic matter content. Consistent with these findings, our results demonstrate that sheep-panel manure at the S2 level (40 kg·m^−2^) significantly enhanced soil moisture content, pH, available nitrogen, available phosphorus, total nitrogen, total phosphorus, and organic matter. The combined application of sheep-panel manure and pelletized organic fertilizer further strengthened these improvements. Among all treatments, S2F2 (40 kg·m^−2^ sheep-panel manure + 3 kg·m^−2^ pelletized organic fertilizer) most markedly enhanced soil moisture content, pH, available nitrogen, available phosphorus, total nitrogen, and organic matter. These results suggest a synergistic interaction between the two amendments. Sheep-panel manure is rich in macro- and micronutrients, can regulate soil pH to create a more favorable environment for plant growth, and provides sustained improvements to soil structure, although its nutrient release is relatively slow [35]. Pelletized organic fertilizer, by contrast, contains balanced nutrients that are more readily available to plants due to processing, with a faster release rate. When applied together, pelletized organic fertilizer can rapidly supply nutrients required during the early stages of plant establishment, whereas sheep-panel manure provides a slow and steady release of nutrients throughout later growth stages. This combined approach is well suited for restoration projects in remote alpine regions where soil-forming processes are slow and repeated interventions are logistically challenging. Chemically, the combined treatment enhances soil pH buffering capacity and promotes nutrient cycling and transformation, particularly nitrogen and phosphorus, thereby substantially increasing soil fertility [36].

### 3.3. Effects of Different Fertilization Treatments on Soil Microbial Biomass

As a reservoir of active nutrients, changes in soil microbial biomass carbon, nitrogen, and phosphorus are sensitive indicators of environmental variation [37]. Microbial biomass not only serves as a key mediator and reserve pool for nutrient transformation in plants but also represents an important indicator of soil development and biochemical functioning [38]. Compared with the application of chemical fertilizers alone, the combined use of chemical fertilizers and organic manure can significantly enhance soil microbial biomass carbon (MBC) [39]. The findings of this study further support this conclusion: the application of sheep-panel manure significantly increased soil MBC, microbial biomass nitrogen (MBN), and microbial biomass phosphorus (MBP), whereas pelletized organic fertilizer alone produced only minor changes. When sheep-panel manure was combined with pelletized organic fertilizer, MBC, MBN, and MBP increased markedly, with the synergistic effect being stronger than the effect of using either amendment alone. The most pronounced improvements were observed under the S2F2 treatment (40 kg·m^−2^ sheep-panel manure + 3 kg·m^−2^ pelletized organic fertilizer). Stoichiometric ratios of microbial biomass are important indicators that reflect shifts in microbial communities. Wang Yutao et al. [40] reported that different fertilization treatments can significantly alter microbial biomass C, N, and P contents, resulting in significant differences in microbial stoichiometry under appropriate fertilization regimes. When the MBC:MBN ratio is below 20, microbial growth is considered carbon-limited; when the ratio exceeds 30, both carbon and nitrogen may inhibit microbial growth; and a ratio of approximately 25 is regarded as optimal for microbial activity [41]. In this study, all MBC/MBN ratios were below 20, indicating that soil microbial growth was limited by both carbon and nitrogen. This pattern was reflected in the pelletized organic fertilizer and combined treatments, where no significant changes were observed. The microbial C:P ratio typically ranges from 7 to 30. Lower values indicate stronger microbial capacity to release phosphorus during organic matter decomposition, thereby contributing to the available phosphorus pool. By contrast, higher C:P ratios suggest that microbes tend to assimilate available phosphorus, potentially increasing competition with plant roots for limited P resources [42]. In this study, only the S2F2 combined treatment displayed MBC/MBP values below 30, suggesting that enhanced microbial mineralization effectively released phosphorus and replenished the soil P pool. Carlos Molina Santiago et al. [43] similarly found that long-term fertilization significantly reduced MBC/MBP relative to unfertilized controls. Our results also showed that the S2 sheep-panel manure treatment significantly reduced MBC/MBN and MBC/MBP compared with the control (S0). This may be attributed to the high carbon content and slow-release nutrients in sheep-panel manure. Its gradual decomposition allows microbes to more efficiently utilize and transform carbon sources while simultaneously supplying additional nitrogen and phosphorus, leading to a more balanced acquisition of essential nutrients and a reduction in microbial biomass carbon relative to nitrogen and phosphorus [44]. Under combined treatments, only S0F2 and S2F0 significantly reduced MBN/MBP. This may be explained by the contrasting nutrient release rates of the two amendments: pelletized organic fertilizer, due to its processed form, releases nutrients rapidly and quickly replenishes soil N and P, whereas sheep-panel manure provides sustained nutrient input through slow mineralization. The observed increases in microbial biomass and improvements in microbial stoichiometry under sheep-panel manure and combined treatments suggest enhanced nutrient cycling efficiency, which is critical for sustaining soil fertility and supporting long-term vegetation recovery in restored mining ecosystems.

### 3.4. Effects of Different Fertilization Treatments on Soil Microbial Community Characteristics

Soil microbial diversity is highly sensitive to changes in soil substrates, and its variation directly reflects the activity level of soil material cycling. Therefore, microbial diversity is often regarded as a key indicator for evaluating soil fertility [45] and plays a crucial role in assessing ecosystem functions and processes [46]. Numerous studies have shown that applying organic fertilizers can modify soil physicochemical properties, influence enzyme activities, and alter microbial biomass and community diversity [47]. Zhang et al. [48] further demonstrated that fertilization not only increases microbial diversity but also reshapes community structure. Other studies have reported that replacing chemical fertilizers with organic amendments, or applying organic fertilizers alone, significantly increases bacterial abundance [49]. Combined application of NPK fertilizers with manure can also substantially enhance bacterial abundance [50]. Similarly, organic fertilizer application has been shown to increase fungal richness and diversity [51]. In high-altitude mining areas, extreme climatic conditions greatly limit both the abundance and diversity of soil microorganisms [52]. Organic fertilizer application can improve soil fertility and restructure microbial community composition [53]. In the present study, the combined application of sheep-panel manure and pelletized organic fertilizer significantly increased bacterial richness and diversity. For fungi, however, only the Shannon index showed a significant improvement. Low temperatures and other environmental stressors in alpine regions strongly suppress microbial proliferation. Bacteria, with their strong adaptive capacity and rapid physiological adjustment, respond swiftly to nutrient enrichment and can overcome environmental constraints more easily than fungi. In contrast, fungal communities, due to their ecological strategies and physiological characteristics, respond more slowly; although organic fertilizer improves their habitat, increases in fungal richness remain limited, and changes are more strongly reflected in community evenness. This aligns with findings from previous work [54], which showed that fungi exhibit higher survival in nutrient-poor reclaimed soils, and that organic fertilizer application exerts limited influence on fungal abundance.

Different fertilization treatments induce distinct responses in soil microorganisms, and different types of organic fertilizers exert varied effects on bacterial and fungal community composition. In this study, although no significant differences were observed in bacterial phyla across treatments, Proteobacteria, Actinobacteria, and Chloroflexi consistently dominated the community. These dominant groups remained stable across treatments. In open-pit mining reclamation studies, researchers have found that as soil nutrients and vegetation recover, the relative abundance of Proteobacteria and Bacteroidetes tends to increase, whereas Actinobacteria and Acidobacteria decline [55]. This may be because Proteobacteria are generally copiotrophic and their abundance responds positively to nutrient enrichment, while many Acidobacteria are oligotrophic and thus decrease when nutrient levels rise [56]. Furthermore, the increase in soil aggregates following combined fertilization may contribute to the reduced abundance of Actinobacteria, which favor more oxygenated conditions [57]. Although pelletized organic fertilizer is fully decomposed, the decomposition of sheep-panel manure consumes substantial soil oxygen, which may inhibit Chloroflexi growth and hinder increases in their abundance [58]. The fluctuating abundances of Sphingomonadaceae and Micrococcaceae across treatments suggest that fertilization had limited overall effects on these groups, potentially because their activity is strongly constrained by low temperatures typical of alpine environments. When temperature declines, their metabolic activity is suppressed, reducing their responsiveness to fertilization. In the fungal community, Ascomycota was the overwhelmingly dominant phylum across all treatments, followed by Basidiomycota, but neither showed consistent variation patterns among treatments. The dominance of Ascomycota may reflect their strong ability to utilize decaying organic substrates [59], while the lack of response in Basidiomycota may be attributed to reduced aeration due to litter cover, which limits their growth. These two phyla are widely recognized as the most abundant and broadly distributed fungal groups in soils [60]. In this study, the genus Chaetomium increased significantly under the S2F1 treatment, likely due to higher soil organic matter and nutrient availability providing abundant carbon sources and energy for its proliferation [61]. PCoA and NMDS analyses based on Bray–Curtis distances showed that neither bacterial nor fungal communities formed distinct clusters under different fertilization treatments, indicating that their evolutionary distances remained relatively small and that fertilization did not fundamentally alter the core structure of microbial communities. The relatively stable microbial community structure in fertilization treatment indicates that nutrient addition mainly enhances microbial activity and functional potential, rather than fundamentally changing community composition, which may help maintain ecological stability in the early recovery stage.

### 3.5. Interactions and Integrated Evaluation of Vegetation, Soil, and Microorganisms

In this study, we analyzed the effects of soil physicochemical properties and microbial biomass on vegetation characteristics under different fertilization treatments during alpine mining-area restoration. The results showed that vegetation height (VH) exhibited the strongest positive correlation under the S2F1 treatment, while vegetation cover (VC) and aboveground biomass (AB) were most strongly correlated under S2F2. Vegetation density (VD) showed the highest correlation under S2F1. These findings highlight that fertilizer combinations influence vegetation traits through distinct pathways. Mantel tests further revealed the ecological linkages between rhizosphere microbial diversity and environmental factors. For bacterial communities, α-diversity was regulated by vegetation density and the MBC/MBN ratio, suggesting that increased vegetation cover can enhance rhizosphere microenvironments and promote microbial community stability [62]. The significant influence of available nitrogen, total nitrogen, total phosphorus, as well as MBC and MBP on bacterial β-diversity indicates that bacteria are highly responsive to nutrient gradients in the soil and may rapidly reorganize their spatial distribution in response to changes in nutrient supply [63]. For fungal communities, α-diversity was primarily affected by microbial biomass nitrogen and available nitrogen, reflecting the key role of fungi in nitrogen cycling and their strong sensitivity to nitrogen availability. Fungal β-diversity was closely correlated with MBC, MBP, pH, and several nutrient indices. This suggests that fungi exhibit pronounced spatial heterogeneity under different soil chemical environments and may adopt varying strategies for utilizing carbon sources and mineral nutrients, thereby fulfilling distinct ecological functions during ecosystem recovery [64].

Regarding fertilization treatments, the weighted evaluation of 15 indicators showed that S2F0 achieved the highest comprehensive score (correlation degree 0.936). This finding underscores the effectiveness of applying a single organic amendment in regulating soil microbial communities and optimizing soil conditions. The result indicates that organic matter inputs can not only improve soil physical and chemical properties—such as reducing bulk density, increasing organic matter content, and enhancing soil moisture—but also stimulate microbial metabolic activity by providing ample organic carbon, thereby influencing microbial community structure and function and ultimately enhancing ecosystem stability [65]. Although the S2F0 treatment achieved the highest comprehensive score in this study, further evaluation of economic costs, labor input, and large-scale field applicability is required before broad implementation in alpine mining restoration projects.

Overall, this study systematically elucidates the interactions among soil microorganisms, vegetation characteristics, and soil physicochemical properties in a high-altitude mining environment. It also clarifies the mechanisms through which different fertilization treatments influence microbial diversity and function. These findings provide both theoretical support and practical guidance for vegetation restoration and soil improvement in degraded mining ecosystems, as well as offer new perspectives for further exploring the complex roles of microorganisms in ecological restoration processes.

Looking ahead, this study also has some limitations. Despite the insights gained, this study was conducted during an early stage of vegetation restoration approximately two years after sowing, which limits the assessment of long-term fertilization effects on vegetation dynamics, soil properties, and microbial communities. The observed positive responses therefore primarily reflect short-term establishment processes and early soil–plant–microbe interactions. Nevertheless, these findings provide important baseline information for understanding initial restoration mechanisms in alpine mining areas and highlight the role of fertilization in facilitating early vegetation establishment. Future studies incorporating longer-term monitoring and continuous fertilization gradients will be essential for evaluating the persistence, stability, and scalability of these restoration strategies and for guiding sustainable ecological restoration in high-altitude mining ecosystems.

## 4. Materials and Methods

### 4.1. Experimental Site Description

The study area is located at Jiangcang No.2 Shaft in the Muli mining region of Qinghai Province (99°59′ E, 38°04′ N) at an elevation of 3816.82 m. The region is situated in a high-altitude alpine zone characterized by extremely harsh climatic conditions and pronounced diurnal temperature fluctuations. During the experimental period, the maximum recorded air temperature reached 19.8 °C, while the minimum temperature dropped to −35.6 °C, with a mean annual temperature of −4.2 °C. Annual precipitation ranged from 473 to 484 mm, whereas the mean annual evaporation reached 1049.9 mm, indicating a strong evaporative demand and frequent soil moisture deficits. The study area is windy throughout the year, with the strongest winds occurring from January to April. During this period, maximum wind speeds exceeded 40 m·s^−1^, and the mean wind speed was 2.9 m·s^−1^. The region also experiences long sunshine duration and intense solar radiation, with annual solar radiation ranging from 610.6 to 721.8 kJ·cm^−2^. The cold season lasts for approximately 7–8 months each year, resulting in an extremely short growing period for pasture vegetation. The original soil types surrounding the Jiangcang mining area are primarily swamp soil and alpine meadow soil, and the native vegetation is dominated by alpine meadows and swamp meadows. Jiangcang No.2 Shaft ceased mining operations in 2014, and ecological restoration activities commenced in 2021. Before the experiment began, the surface layer of the spoil heap consisted mainly of coal gangue and various types of fragmented rock. The initial soil physicochemical properties were as follows: total nitrogen 1.165 g·kg^−1^, total phosphorus 0.91 g·kg^−1^, available nitrogen 0.018 g·kg^−1^, available phosphorus 0.0074 g·kg^−1^, organic matter 93.33 g·kg^−1^, moisture content 13.23%, bluk density 16.08 g·cm^−3^ and pH 8.46.

### 4.2. Determination of Research Subjects

**Figure 10:**
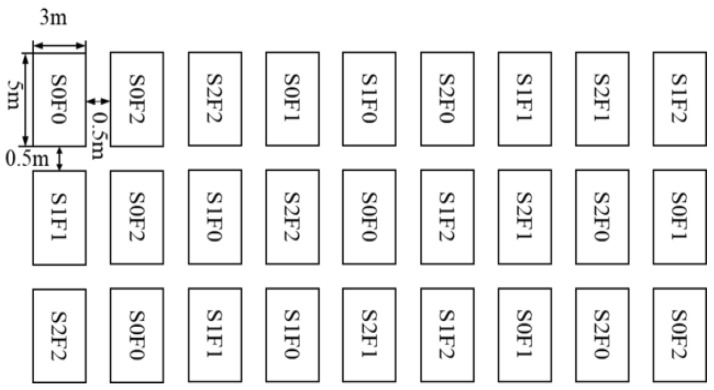
Experimental setup diagram.

Prior to sowing in June 2021, stones were removed from the site, and the surface of the spoil-heap platform was leveled. Experimental plots were arranged following a randomized block design. Drainage ditches were constructed around the experimental field to prevent waterlogging. For each plot, fertilizers corresponding to the assigned treatment levels were thoroughly mixed with the upper 15 cm of spoil soil, and all fertilizers were applied once prior to sowing in June 2021, at the time of plot establishment. A mixture of four local forage species—Qinghai Festuca sinensis, Tongde Cleistogenes squarrosa, Qinghai cold-season Poa pratensis, and Qinghai grassland Poa pratensis—was sown at a mass ratio of 1:1:1:1. The seeding rate was 18 g·m^−2^, with a sowing depth of 1 cm. All plots were covered with non-woven fabric after seeding. The application rates of sheep-panel manure and pelletized organic fertilizer were determined based on commonly adopted practices in alpine grassland restoration, local resource availability, and the contrasting physical and chemical properties of the two amendments. Sheep-panel manure (organic matter content: 429.75 g·kg^−1^; moisture: 37.19%; N + P_2_O_5_+ K_2_O content 37.62 g·kg^−1^) was applied at three levels: 0 kg·m^−2^ (S0), 20 kg·m^−2^ (S1), and 40 kg·m^−2^ (S2). Pelletized organic fertilizer—provided by Qinghai Plateau Difeng Fertilizer Co., Ltd. (Golmud, China) (organic matter ≥ 450 g·kg^−1^, moisture ≤ 30%, N + P_2_O_5_+ K_2_O ≥ 50 g·kg^−1^)—was applied at three levels: 0.00 kg·m^−2^ (F0), 1.50 kg·m^−2^ (F1), and 3.00 kg·m^−2^ (F2). The combination of the two factors resulted in nine treatment levels: S0F0, S0F1, S0F2, S1F0, S1F1, S1F2, S2F0, S2F1, and S2F2 (Figure 10). Each treatment was replicated three times, with each plot measuring 3 m × 5 m, yielding a total of 27 plots. Detailed fertilizer combinations are presented in Table 3.

### 4.3. Soil Sampling and Determination

In August 2023, vegetation surveys and soil sampling were conducted in each plot. Three 1 m × 1 m quadrats were established within each plot to measure plant height, canopy cover, plant density, and aboveground biomass. Plant height, representing the height of the herbaceous layer, was measured using a measuring tape on 20 randomly selected individuals. Canopy cover was estimated using the point-intercept method. Aboveground biomass was determined by clipping all aboveground plant material at ground level within each quadrat, followed by oven-drying at 65 °C in the laboratory and weighing. Within the same quadrats used for vegetation measurements, soil samples (0~15 cm depth) were collected using a soil auger. Four subsamples were randomly taken from each quadrat and homogenized to form one composite sample, with each quadrat serving as one replicate. A small portion of the homogenized soil was placed into cryogenic tubes, immediately transferred to a portable low-temperature storage device, and subsequently stored at −80 °C for microbial analyses. The remaining soil was air-dried in the laboratory for soil fertility measurements. Soil moisture content and bulk density were determined using the cutting-ring oven-drying method. Soil pH was measured in a 1:5 soil-to-water suspension using a pH meter. Soil organic matter was quantified by the potassium dichromate (K_2_Cr_2_O_7_) oxidation method. Total phosphorus was determined using the ammonium molybdate spectrophotometric method. Available nitrogen and total nitrogen were measured by the alkaline hydrolysis diffusion method and the Kjeldahl digestion method, respectively. Available phosphorus was determined using the molybdenum blue colorimetric method [66]. Soil microbial biomass carbon (MBC) was measured using the chloroform fumigation K_2_SO_4_ extraction method followed by TOC analysis. Microbial biomass nitrogen (MBN) was determined through chloroform fumigation K_2_SO_4_ extraction combined with the Kjeldahl method. Microbial biomass phosphorus (MBP) was measured using the chloroform fumigation −0.5 mol/L NaHCO_3_ extraction method [67].

### 4.4. DNA Extraction, PCR Amplification and High-Throughput Sequencing

Total microbial genomic DNA was extracted from soil samples using the E.Z.N.A.^®^ Soil DNA Kit (Omega Bio-tek, Norcross, GA, USA) according to the manufacturer’s instructions. DNA quality and concentration were assessed by 1.0% agarose gel electrophoresis and a NanoDrop 2000 spectrophotometer (Thermo Scientific, Waltham, MA, USA), and the extracted DNA was stored at −80 °C until further analysis. The V3–V4 hypervariable region of the bacterial 16S rRNA gene was amplified using the primer pair 338F (5′-ACTCCTACGGGAGGCAGCAG-3′) and 806R (5′-GGACTACHVGGGTWTCTAAT-3′) on a T100 Thermal Cycler PCR system (Bio-Rad, Hercules, CA, USA). Each PCR reaction was performed in a total volume of 20 μL, containing 4 μL of 5× FastPfu buffer, 2 μL of 2.5 mM dNTPs, 0.8 μL of each primer (5 μM), 0.4 μL of FastPfu DNA polymerase, 10 ng of template DNA, and nuclease-free water. The PCR amplification conditions were as follows: initial denaturation at 95 °C for 3 min; followed by denaturation at 95 °C for 30 s, annealing at 55 °C for 30 s, and extension at 72 °C for 45 s; with a final extension at 72 °C for 10 min, and termination at 4 °C. PCR products were extracted from 2% agarose gels, purified using a PCR purification kit (YuHua, Shanghai, China) according to the manufacturer’s instructions, and quantified using a Qubit 4.0 fluorometer (Thermo Fisher Scientific, Waltham, MA, USA).

### 4.5. Sequencing Process and Bioinformatics Approaches

Raw FASTQ files were demultiplexed using in-house Perl scripts, followed by quality filtering using fastp version 0.19.6 and sequence merging using FLASH version 1.2.7. The following criteria were applied: (i) Reads were truncated at any site with an average quality score < 20 over a 50 bp sliding window; truncated reads shorter than 50 bp, as well as reads containing ambiguous bases, were discarded. (ii) Only overlapping sequences with an overlap length greater than 10 bp were assembled, with a maximum mismatch ratio of 0.2 allowed in the overlap region, and non-assembled reads were removed. (iii) Sequences were assigned to samples based on barcodes and primers, with exact barcode matching and up to two nucleotide mismatches permitted in primer matching, and sequence orientations were adjusted accordingly. High-quality sequences were clustered into operational taxonomic units (OTUs) at 97% sequence similarity using UPARSE version 7.1, and the most abundant sequence within each OTU was selected as the representative sequence. Taxonomic assignment of bacterial OTUs was performed using the SILVA rRNA gene database, while fungal OTUs were classified against the UNITE database. To minimize the influence of sequencing depth on α- and β-diversity analyses, the number of 16S rRNA gene sequences per sample was rarefied to 20,000, resulting in an average Good’s coverage of 99.09%. After quality control, the number of effective reads per sample was sufficient for downstream diversity and community composition analyses, and sequencing depth adequacy was evaluated based on OTU richness stabilization and coverage values. Purified amplicons were pooled in equimolar concentrations and subjected to paired-end sequencing on the Illumina PE300/PE250 platform (Illumina, San Diego, CA, USA) according to the standard protocols of Majorbio Bio-Pharm Technology Co., Ltd. (Shanghai, China).

### 4.6. Statistical Analysis

In this study, SPSS 27.0 was used to conduct two-way analysis of variance (ANOVA) on plant and soil variables under different fertilization treatments. Prior to ANOVA, data normality was assessed using the Shapiro–Wilk test, and homogeneity of variances was evaluated using Levene’s test. Principal coordinates analysis (PCoA) based on the Bray–Curtis distance was applied to assess similarities in bacterial and fungal community structures among samples, and PERMANOVA was further employed to test the significance of differences in microbial community composition among treatment groups. Using an online analytical platform (https://www.omicstudio.cn/tool/109, accessed on 15 March 2024), Pearson correlation coefficients were calculated to construct heatmaps illustrating the relationships among plant community characteristics, soil physicochemical properties, microbial biomass, and microbial community attributes. A comprehensive evaluation of optimal application rates was performed using SPSSAU [68]. All remaining figures were generated using Origin 2022 software.

## 5. Conclusions

This study demonstrated that the combined application of sheep-panel manure and commercial organic fertilizer exerted significant effects on vegetation characteristics, soil physicochemical properties, soil microbial biomass, and microbial community structure in a high-altitude mining area. Fertilization markedly enhanced vegetation height, canopy cover, plant density, and aboveground biomass, with the strongest improvements observed under the S2F2 and S2F1 treatments. Compared with other treatments, S2F2 significantly increased soil moisture, pH, soil organic matter, available nitrogen, available phosphorus, total nitrogen, and microbial biomass C, N, and P. The S0F2 treatment significantly increased bacterial Observed Richness, Shannon, and Chao1 indices, and also enhanced fungal Observed Richness and Chao1. Vegetation height and density showed the strongest positive correlations under S2F1, while canopy cover and aboveground biomass exhibited the strongest correlations under S2F2. Based on the weighted evaluation of 15 indicators, the S2F0 treatment (40 kg·m^−2^ sheep-panel manure + 0 kg·m^−2^ pelletized organic fertilizer) achieved the highest comprehensive score (correlation degree: 0.936), indicating its notable advantage in regulating soil microbial communities and improving soil conditions in high-altitude mining ecosystems.

## Figures and Tables

**Table 1:** Correlation coefficient results.

	Indicators	S0F0	S0F1	S0F2	S1F0	S1F1	S1F2	S2F0	S2F1	S2F2
Soil Physicochemical Properties	pH	1.000	0.955	0.953	0.935	0.933	0.941	0.944	0.933	0.929
	AN	0.953	0.951	0.954	0.958	0.959	0.981	1.000	0.986	0.983
	AP	0.953	0.951	0.955	0.958	0.960	0.982	1.000	0.987	0.984
	TN	0.956	0.951	0.963	0.957	0.962	0.975	1.000	0.994	0.994
	TP	0.958	0.954	0.955	0.956	0.965	0.981	1.000	0.986	0.982
	SOM	1.000	0.571	0.526	0.467	0.454	0.387	0.333	0.344	0.346
Soil microbial biomass	MBC	0.385	0.583	0.629	0.702	0.722	0.857	1.000	0.957	0.950
	MBN	0.784	0.805	0.797	0.885	0.890	0.858	0.882	0.984	1.000
	MBP	0.905	0.926	0.930	0.938	0.938	0.947	1.000	0.986	0.991
Bacterial community	Shannon index	1.000	0.942	0.940	0.914	0.911	0.919	0.917	0.905	0.902
	Simpson index	0.967	0.959	0.961	0.962	0.963	0.983	1.000	0.986	0.983
	pielou index	0.965	0.958	0.960	0.961	0.962	0.983	1.000	0.986	0.983
Fungal community	Shannon index	1.000	0.963	0.967	0.953	0.952	0.964	0.971	0.950	0.952
	Simpson index	0.967	0.958	0.961	0.962	0.963	0.983	1.000	0.985	0.983
	pielou index	0.962	0.956	0.959	0.961	0.962	0.983	1.000	0.986	0.983

**Table 2:** Correlation degree results.

Evaluation Criteria	Relevance	Ranking
S0F0	0.917	3
S0F1	0.892	8
S0F2	0.894	7
S1F0	0.898	6
S1F1	0.900	5
S1F2	0.915	4
S2F0	0.936	1
S2F1	0.930	2
S2F2	0.930	2

**Table 3:** Fertilization treatments under different conditions.

Treatment	Sheep Board Manure (kg·m^−2^)	Organic Fertilizer (kg·m^−2^)
S0F0	0	0.00
S0F1	0	1.50
S0F2	0	3.00
S1F0	20	0.00
S1F1	20	1.50
S1F2	20	3.00
S2F0	40	0.00
S2F1	40	1.50
S2F2	40	3.00

## Data Availability

The original contributions presented in this study are included in the article. Further inquiries can be directed to the corresponding author.

## Supplementary Materials

The following supporting information can be downloaded at https://www.mdpi.com/article/10.3390/plants15040569/s1. Table S1: Two-way ANOVA of vegetation characteristics under different fertilization treatments. Values shown as 0.000 represent *p*-values smaller than 0.001 due to rounding by the statistical software, the same below. Table S2: Two-way ANOVA of soil physicochemical properties under different fertilization. Table S3: Two-way ANOVA of soil microbial biomass carbon, nitrogen, phosphorus, and their stoichiometric ratios under different fertilization treatments.
