# Supplementary material for: Responses of Restored Vegetation Communities, Soil Properties, and Microbial Composition to Different Fertilization Treatments in an Alpine Mining Area
Source: Plants (Basel). 2026 Feb 11;15(4):569. doi: 10.3390/plants15040569 (PMC12944704; doi:10.3390/plants15040569)
Supplement: Supplementary file 1 [file plants-15-00569-s001.zip › plants-4108253-supplementary.pdf]

Table S1. Two-way ANOVA of vegetation characteristics under different fertilization treatments. Values shown as 0.000 represent p-values smaller than 0.001 due to rounding by the statistical software, the same below.

Treatment	*df*	Vegetation height(cm)		Vegetation coverage(%)		Vegetation density(plants·m^{-2})		Above-ground biomass(g·m^{-2})	
		F	*P*	*F*	*P*	*F*	*P*	*F*	*P*
S	2	12.209	0.000	50.818	0.000	10.462	0.001	13.317	0.000
F	2	8.686	0.002	10.886	0.001	4.761	0.022	2.022	0.161
S*F	4	10.503	0.000	5.908	0.003	2.550	0.075	2.166	0.114

Table S2. Two-way ANOVA of soil physicochemical properties under different fertilization.

Indicators	*df*	S	F	S*F
		2	2	4
Moisture content/(g·cm^{-3})	*F*	321.725	12.073	9.407
	P	0.000	0.000	0.000
Bluk density/(g·cm^{-3})	*F*	783.981	80.695	98.075
	P	0.000	0.000	0.000
pH	*F*	25.087	9.494	2.786
	P	0.000	0.002	0.058
Available nitrogen/(mg·kg^{-1})	*F*	40.184	3.587	1.968
	P	0.000	0.049	0.143
Available phosphorus/(mg·kg^{-1})	*F*	74.325	22.195	4.196
	P	0.000	0.000	0.014
Total nitrogen/(g·kg^{-1})	*F*	70.160	5.049	0.605
	P	0.000	0.018	0.664
Total phosphorus/(g·kg^{-1})	*F*	50.930	3.058	1.807
	P	0.000	0.072	0.171
Organic matter/(g·kg^{-1})	*F*	38.474	3.771	0.813
	P	0.000	0.043	0.533

Table S3. Two-way ANOVA of soil microbial biomass carbon, nitrogen, phosphorus, and their stoichiometric ratios under different fertilization treatments.

Treatment	*df*	MBC		MBN		MBP		MBC/MBN		MBC/MBP		MBN/MBP	
		F	*P*	*F*	*P*	*F*	*P*	*F*	*P*	*F*	*P*	*F*	*P*
S	2	2075.819	0.000	369.641	0.000	496.969	0.000	8.509	0.003	2.768	0.001	2.662	0.097
F	2	82.352	0.000	7.298	0.005	31.787	0.000	1.669	0.216	0.147	0.090	0.185	0.833
S*F	4	22.799	0.000	2.161	0.115	12.896	0.000	6.907	0.001	0.930	0.864	2.994	0.047
